# Studying Cationic Liposomes for Quick, Simple, and Effective Nucleic Acid Preconcentration and Isolation

**DOI:** 10.1021/acs.analchem.4c05936

**Published:** 2025-01-29

**Authors:** Rahel Gruenberger, Changyoon Baek, Clemens Spitzenberg, Junhong Min, Antje J. Baeumner

**Affiliations:** † Institute of Analytical Chemistry, Chemo- and Biosensors, University of Regensburg, Universitaetsstr. 31, Regensburg 93053, Germany; ‡ School of Integrative Engineering, Chung-Ang University, Heukseok-Dong, Dongjak-Gu, Seoul 06974, Republic of Korea

## Abstract

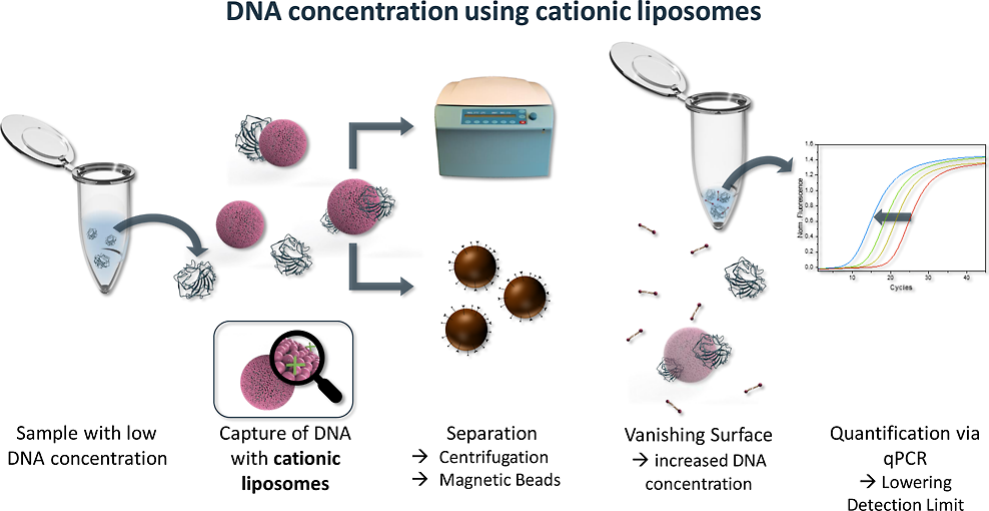

To ensure high quality of food and water, the identification of traces of pathogens is mandatory. Rapid nucleic acid-based tests shorten traditional detection times while maintaining low detection limits. Challenging is the loss of nucleic acids during necessary purification processes, since elution off solid surfaces is not efficient. We therefore propose the development of a vanishing surface strategy in which cationic liposomes efficiently capture nucleic acids. A lipoplex is formed that can be easily centrifuged down and washed if needed. Adding the lipoplex to detergent solutions or nucleic acid amplification reactions dissolves the liposomes, releasing 100% of the nucleic acid into the reaction. After initial protocol optimization, it was applied to isolate DNA from *Escherichia coli*, *Staphylococcus aureus,* and adenovirus in buffer followed by qPCR detection. This enabled the detection of these pathogens down to concentrations of 1 CFU or 1 PFU/mL, respectively. Comparing it to a standard commercial DNA extraction kit, it was superior, as evidenced by lower Ct-values in the qPCR for all pathogen concentrations. Scaling up to larger volumes, samples containing bacteria were first concentrated through nitrocellulose filters (pore size = 0.45 μm). Tap water, lake water, and rinse water of fresh produce were investigated, leading to relevant limits of detection of 100 CFU in 100 mL of tap water, 1000 CFU in 100 mL of lake water, and 100 CFU in 10 g of iceberg lettuce, respectively. Since the liposome protocol is a homogeneous, simple incubation step, it is a valuable alternative to standard commercial nucleic acid extraction kits.

## Background

Nucleic acid extraction is integral to molecular biology, as it enables the isolation and purification of DNA from various real samples. It is hence ubiquitous in biological and medical research, in routine diagnostics of medical and environmental samples.^1−3^ Especially in the detection of pathogens in water and food samples to prevent water and foodborne diseases, the ability to efficiently extract, purify, and concentrate DNA from limited starting material is crucial for accurate molecular detection.^4,5^

However, conventional methods, such as solid-phase extraction (SPE), can be challenging at low DNA concentrations. Solid-phase purification is usually carried out using a centrifuge column.^6^ The solid carriers used include silica matrices, glass particles, diatomaceous earth, and anion exchange carriers. The four most important steps in SPE are cell lysis, adsorption of nucleic acids, washing, and elution,^7^ bearing two inherent loss mechanisms. First, DNA adsorption onto the column may be inefficient, and second, the adsorbed DNA may not be efficiently eluted from the column. The elution of nucleic acids from high-affinity matrices, such as cellulose or synthetic resins, is difficult and can therefore lead to low yields. Most importantly, the SPE using silica columns does not work optimally when trying to bind and release minute amounts of total DNA. Most commercial kits solve this problem by adding exogenous DNA to artificially increase the total DNA load.^8−10^ However, this approach is not considered ideal, as adding reagents increases the cost and complexity of testing, gives room for contamination, and is not applicable to all subsequent quantification strategies.

In such cases, an alternative method is needed that offers a higher efficiency. Newer approaches use magnetic nanoparticles for nucleic acid extraction. By modifying the surface, the affinity of the beads consisting of iron oxide particles to the DNA is increased.^2^ These modifications can vary from a silica coat, specific oligonucleotides, or positively charged polymers.^11,12^ Silica-coated magnetic beads have been shown to be successfully integrated into microfluidic systems for point-of-care isolation and detection of nucleic acids.^13^ Another approach for point-of-care extraction would be a paper-based process, e.g., using chitosan-treated paper or charge-switchable polymers that bind or release DNA depending on the pH value.^14,15^

This study investigates a DNA extraction method based on the interaction between cationic liposomes and polyanionic DNA to detect pathogens at a low concentration without time-consuming enrichment steps. Quantitative polymerase chain reaction (qPCR) is used to best identify differences in isolation and elution efficiencies. Liposomes are spherical nanovesicles with a lipid bilayer that encloses an aqueous cavity.^16^ The surface of the lipid bilayer can be modified to obtain specific functionality, leading to numerous applications in the fields of drug and gene delivery, vaccination, chromatography, and biosensing.^17−21^ By using lipids with charged head groups, the ζ-potential can be shifted to positive or negative.^22^

Cationic lipids have become key players in the field of genetic delivery studies. These versatile vectors self-assemble with anionic nucleic acids and form protective nano- or microparticles known as lipoplexes. Cationic lipids typically feature a cationic headgroup covalently bound through a linker to a hydrophobic tail.^23^ The first chemically synthesized cationic lipid was *N*-(1-(2,3-dioleyloxy)propyl)-*N*,*N*,*N*-trimethylammonium chloride (DOTMA) in 1987.^24^ Since then, a wide variety of lipids were synthesized by modifying the individual components to enhance transfection.^25^ An example of a cationic lipid is 1,2-dipalmitoyl-*sn*-glycero-3-ethylphosphocholine (EDPPC), which is chemically stable and forms stable liposomes, that enclose DNA between the lipid bilayers and thus form lipoplexes.^26^

Alternatively, when cationic liposomes are added to DNA, stable lipoplexes are formed into larger conglomerates that can be easily centrifuged off and thus separated from the matrix. Subsequently, such lipoplexes can directly be added to amplification reactions such as a PCR mixture without the need for elution and without interfering with the reaction. By using a detergent as an additive in the PCR mixture, the liposomes are easily lysed, leading to a dissolution of the lipoplexes, releasing the DNA free into solution to serve as the substrate in the amplification reaction.

This DNA extraction method is evaluated against commercially available kits that utilize SPE, particularly for low pathogen concentrations. Furthermore, DNA extraction via liposomes is employed in the sample preparation of real samples from the water and food sector. Typically, large sample volumes are used so that bacteria are first concentrated via filtration, then lysed, subjected to the liposome concentration step, and finally quantified via qPCR.

## Experimental Section

### Chemicals and Materials

Tris(hydroxymethyl)-aminomethane was purchased from Affymetrix USB. Lysogen broth (LB) was purchased from Alfa Aesar. 1,2-Dipalmitoyl-*sn*-glycero-3-phosphatidylcholine (DPPC), 1,2-dipalmitoyl-*sn*-glycero-3-phosphoethanolamine-*N*-(biotinyl) (sodium salt) (DPPE-Biotin), cholesterol, the extrusion kit, and membranes were purchased from Avanti Polar Lipids. Octyl-β-d-glucopyranoside (OG), sample bags (Rotilabo), sodium chloride, and sodium hydroxide (1 M) were bought from Carl Roth. EDPPC was bought from Cayman Chemical Company. Sulfuric acid (98%) and hydrogen peroxide (35%) were purchased from DAEJUNG chemical and materials. Glass beads were sourced from Daihan Scientific in Korea. *Escherichia coli* K12 (*E. coli*) and *Staphylococcus aureus* (*S. aureus*) were purchased from the DSMZ-German Collection of Microorganisms and Cell Cultures. Human adenovirus type 5 and HEK 293 cells were acquired from Professor Seol Dae-Woo (College of Pharmacy, Chung-Ang University, Republic of Korea). Syringe filters (glass wool, pore size: 2 μm, Merck Millex) were purchased from Fisher Scientific. Dulbecco’s modified Eagle medium (DMEM) and fetal bovine serum (FBS) were purchased from Gibco, Thermo Fisher Scientific. The PCR tubes were obtained from Kisker Biotech. Hydrochloric acid (1 M), nylon membrane (0.20 μm, hydrophilic), sodium azide, and sucrose were bought from Merck. Syringes (20 mL, Norm-Ject) were purchased from MSG. Nucleic acid extraction and purification kits (QIAamp DNA mini kit, QIAamp MinElute Virus Spin kit) were purchased from Qiagen. DNA LowBinding Cups were purchased from Sarstedt. Ethylenediaminetetraacetic acid disodium salt dihydrate (EDTA), GenElute Bacterial Genomic DNA Kit, Tris–EDTA buffer (TE buffer), and Tween80 were purchased from Sigma-Aldrich. Dialysis membranes were obtained from spectrum laboratories (spectra/por 4 with a MWCO of 12–14 kDa). TB Green Premix Ex Taq (Tli RNaseH Plus) was purchased from Takara. DNase free water (DFW), DNA primers (10 μM), and magnetic beads conjugated to streptavidin (Dynabeads MyOne Streptavidin C1, Invitrogen) were purchased from Thermo Fisher Scientific. 4-(2-Hydroxyethyl) piperazine-1-ethanesulfonic acid (HEPES), agar addition, and filter membranes (cellulose nitrate, pore size: 0.45 μm, Sartorius) were purchased from VWR.

Lake water was sampled from the University Lake at the University of Regensburg, Germany. Tap water was sampled from the supply system of the University Regensburg, Germany. Iceberg lettuce was purchased from a local grocery store.

For additional information about buffer and medium compositions, see Supporting Information.

### Liposome Synthesis

Liposomes were synthesized using the reverse-phase evaporation method as described previously.^27^ The composition of cationic liposomes has been optimized by Hofmann et al. previously.^28^

The encapsulant (4.5 mL) was prepared by dissolving NaCl (300 mM) in HEPES (20 mM, pH 7.5). The solution was placed in a heat bath at 60 °C. Lipids (Table S2) containing 18 Mol% EDPPC were dissolved in chloroform (3 mL) and methanol (0.5 mL). This mixture was then sonicated for 1 min at 60 °C. Afterward, the preheated encapsulant (2 mL, 300 mM, pH 7.5) was added. Subsequently, the solution was sonicated again for 4 min at 60 °C. The organic solvents were evaporated at a rotary evaporator (LABOROTA 4001) at 60 °C. The pressure was reduced stepwise (900 mbar for 10 min, 850 mbar for 5 min, 800 mbar for 5 min, and 780 mbar for 20 min). After the mixture was vortexed for 30 s, another 2 mL of the encapsulant was added, followed by another step of vortexing. Residual organic solvents were removed at 60 °C (750 mbar for 20 min, 600 mbar for 5 min, 500 mbar for 5 min, and 400 mbar for 20 min). The solution was extruded at 60 °C through polycarbonate membranes (1 and 0.4 μm) by forcing the solution through each membrane 21 times. Excess sodium chloride was removed by size exclusion chromatography, using a Sephadex G-50 column. The fractions of chromatography were collected and divided into highly and medium-concentrated fractions. Finally, the fractions were purified by dialysis against HSS buffer (HEPES, sodium chloride, and sucrose buffer) at room temperature for approximately 18 h. The buffer was exchanged two times.

### Characterization of Liposomes

Lipid concentrations were determined via optical emission spectroscopy with inductively coupled plasma (ICP-OES) (SpectroBlue TI/EOP) from SPECTRO Analytical Instruments GmbH. The liposome solution was diluted 1:100 in 0.5 M HNO_3_. Phosphorus standards diluted in 0.5 μM HNO_3_ from 0 to 100 μM were used for calibration. Phosphorus was detected at 177.495 nm. With this method, the phospholipid concentration was determined. The total lipid (tL) concentration was calculated with the phospholipid concentration and the lipid composition used in the synthesis.

Size and ζ-potential determinations were performed via dynamic light scattering (DLS) on a Zetasizer Nano-ZS from Malvern Panalytical. The liposomes were diluted by 1:100 in HSS buffer. For size measurements, poly(methyl methacrylate) (PMMA) semimicro cuvettes and for ζ-potential measurements disposable, folded capillary cells (Malvern Panalytical) were used. The measurement temperature was set to 25 °C. As settings, a refractive index *n*_D_(20) of 1.34, a material absorbance of zero, a dispersant viscosity of 1.1185 kg m^–1^ s^–1^, and a dielectric constant of 78.5 were selected. An equilibration time of 60 s was applied before each measurement.

### Cell Culture

Cultures were made in LB medium by inoculating 10 mL of medium with one colony or 10 μL of a liquid culture of the respective bacteria strain and cultured overnight at 37 °C. The optical density of a 1:20 dilution of the culture in LB medium was measured at 600 nm. Pure LB medium was used as the blank. For DNA extraction experiments, the overnight culture was diluted in TE buffer to the desired concentration and then added to the sample matrix.

### Adenovirus Preparation

Human adenovirus type 5 and HEK 293 cells were cultured in DMEM supplemented with 10% FBS. The virus was propagated in HEK 293 cells for 2 days and then harvested via freeze–thaw cycles. The harvested virus was stored at 4 °C until use in the experiments.

### DNA Extraction and Purification Using Extraction Kits

DNA purification for the recovery rate determination was done using the GenElute Bacterial Genomic DNA Kit from Thermo Fisher for “Gram-negative bacteria”. The purity of the extracted DNA was analyzed with a BioSpectrometer from Eppendorf by Nanodrop measurement.

For extraction efficiency comparison experiments, DNA purification was done using QIAGen Kits. For virus experiments, a QIAamp MinElute Virus Spin Kit was used. For bacterial experiments, the QIAamp DNA Mini Kit was used.

### Real Sample Pretreatment

To remove larger particles or inhibitors, the samples are prefiltered with a syringe filter (glass wool, pore size = 2 μm).

Bacteria were concentrated by filtration through a cellulose nitrate membrane (pore size of 0.45 μm). To resolubilize the bacteria, the filter was soaked in TE buffer (2 mL) and tapped for 60 s 500 μL of the solution were taken for DNA extraction.

### Bead Preparation for Bead Beating

Using a piranha solution (composed of 98% H_2_SO_4_: 35% H_2_O_2_ in a ratio of 3:1), 70–100 μm glass beads underwent a 30 min cleaning process and were subsequently washed with deionized water. Following cleaning, the beads were dried at 70 °C for 3 h.

### Pathogen Lysis

Pathogens were lysed using two different methods. *S. aureus* and adenovirus were lysed using bead beating. During bead beating, pathogen solutions in TE buffer (500 μL) were vortexed for 3 min at 3000 rpm with glass beads (approximately 400 mg). The supernatant (350 μL) with the lysed pathogens was used for DNA extraction.

The ultrasound device Bioruptor from Diagenode lyses bacteria with a frequency of 20 kHz and an intensity of 320 W. A sonication time of 5 min with 30 s on- and off-intervals was applied for *E. coli* bacteria. The samples were rotated during the off phases to ensure a homogeneous power density distribution. The water in the bath was cooled with ice. The complete volume (500 μL) was used for DNA extraction.

### Liposome Assay for DNA Extraction and Preconcentration Using Centrifugation

The liposome assay was performed using either already extracted DNA or lysed pathogens. The procedure consisted of adding cationic liposomes to a DNA-containing matrix (500 μL). The liposome concentration was adjusted to 10 μM. The sample was then incubated at 30 °C for 15 min using a thermo shaker (300 rpm). During this time, the liposomes bound the DNA and formed the lipoplex. After centrifugation at 15,000 g for 10 min, the supernatant was completely removed while keeping the lipoplex in precipitate. Then the lipoplex was resolubilized in the “20 μL”-PCR mix and the DNA content was measured using real-time PCR. Data on parameter optimization can be found in the Supporting Information (Figures S1–S5).

### MagBead Assay for DNA Extraction and Preconcentration

Magnetic beads (magBeads) (Dynabeads MyOne Streptavidin C1) (5 μL per sample) were washed twice with 10 times the original volume of binding and washing buffer (B&W buffer) and once with 10 times the original volume of TE buffer. The magBeads were resuspended in their original volume in TE buffer. The capture probe was obtained by incubating magBeads and biotinylated cationic liposomes in a volume ratio of 5:1 magBeads to liposomes for 45 min at room temperature. Unbound liposomes were eliminated by a washing step using TE buffer (100 μL). The capture probe was resuspended in the original volume of the magBeads. The capture probe (5 μL) was added to DNA containing matrices (150 μL) and incubated for 45 min at 30 °C using a thermo shaker (300 rpm). Afterward the capture probe was separated using a magnet, and the supernatant was removed. The beads were resuspended in a detergent solution (5 μL) containing OG (10 mM) and incubated for 10 min at 90 °C. The concentrated DNA solution without magBeads was added to a “15 μL”-PCR mix and the DNA content was measured using real-time PCR.

### Real-Time PCR

Real-time PCR measurements were performed using the qPCR devices Qiagen Rotor-Gene Q or Light Cycler 96. For reference measurements, an “18 μL”-PCR mix was prepared into which the DNA (2 μL) was pipetted in directly. For samples, where the liposome assay was performed, a “20 μL”-PCR mix was used and pipetted onto the lipoplex. For samples where the magBead assay was performed, a “15 μL”-PCR mix was prepared, and the DNA diluted in a detergent solution (5 μL) was added.

The PCR mix contained TB Green Premix Ex Taq, Primers, DFW, and Tween80 (4 wt %). PCR-mix compositions can be found in Table 1.

**Table 1:** Composition of the “15 μL”-PCR mix, “18 μL”-PCR mix, and the “20 μL”-PCR mix

	DFW	Tween80 (4 wt %)	TB green premix Ex Taq	primer
“15 μL”-PCR mix	4 μL	-	10 μL	0.5 μL of each primer (forward and reverse)
“18 μL”-PCR mix	2 μL	5 μL		
“20 μL”-PCR mix	4 μL			

Information regarding the measurement settings and primer sequences can be found in the Supporting Information (Tables S3 and S4).

### Calculation of the Recovery Rate

To calculate the amount of DNA recovered by the liposome assay, a calibration curve was required. For this purpose, a series of DNA dilutions was measured with known concentration. The logarithm of the DNA quantity was plotted against the Ct-value. Through this calibration curve, the DNA content could be determined with eq 1
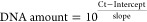
1

To determine the extraction efficiency of the liposome assay, the recovered DNA amount by the assay (calculated with eq 1) was divided by the DNA amount that was originally applied (see eq 2)
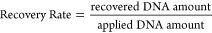
2

## Results and Discussion

For the development of a new platform technology for nucleic acid isolation, cationic liposomes were synthesized, optimized, and applied to real samples. Following a previously developed protocol, liposomes using 18 Mol% EDPPC were synthesized^28^ and fully characterized with respect to tL amount, size, and ζ-potential (see Table S2) to allow for reliable comparison between synthesis lots. After incubation of liposomes and DNA-containing samples, separation of the lipoplex from the remaining solution was achieved either via quick centrifugation or through magnetic separation. The second separation method employs the binding of streptavidinylated magnetic particles and biotinylated cationic liposomes, thereby facilitating the point-of-care separation of DNA.

### Liposome Assay Recovery Rate Determination

**Scheme 1:**
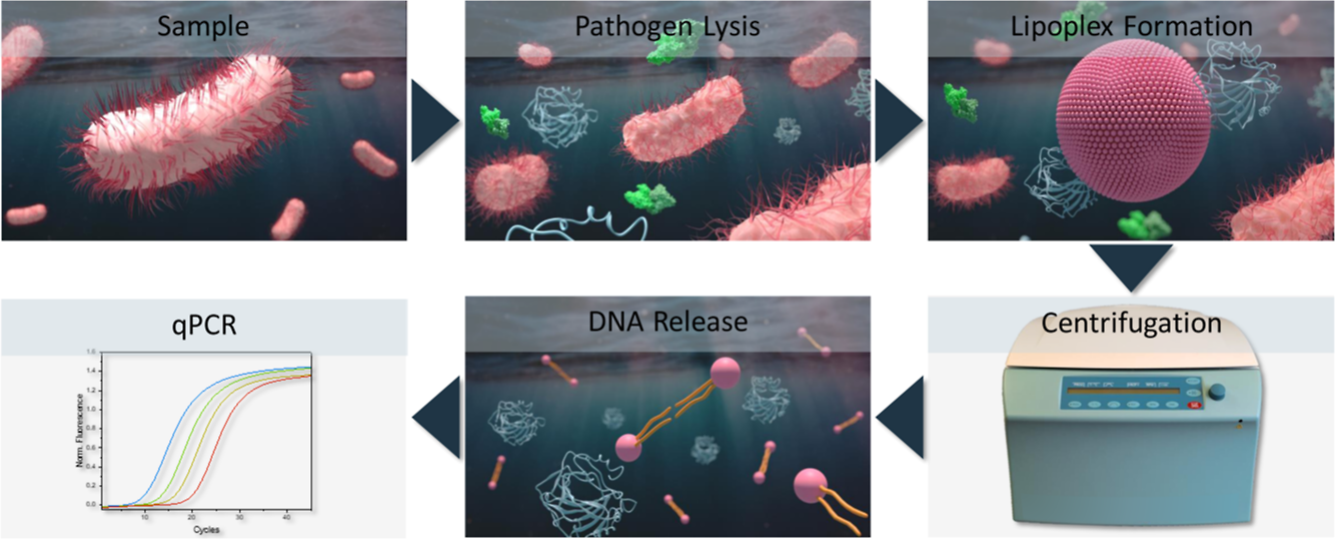
Schematic of the Working Principle of the Liposome Assay

**Figure 1:**
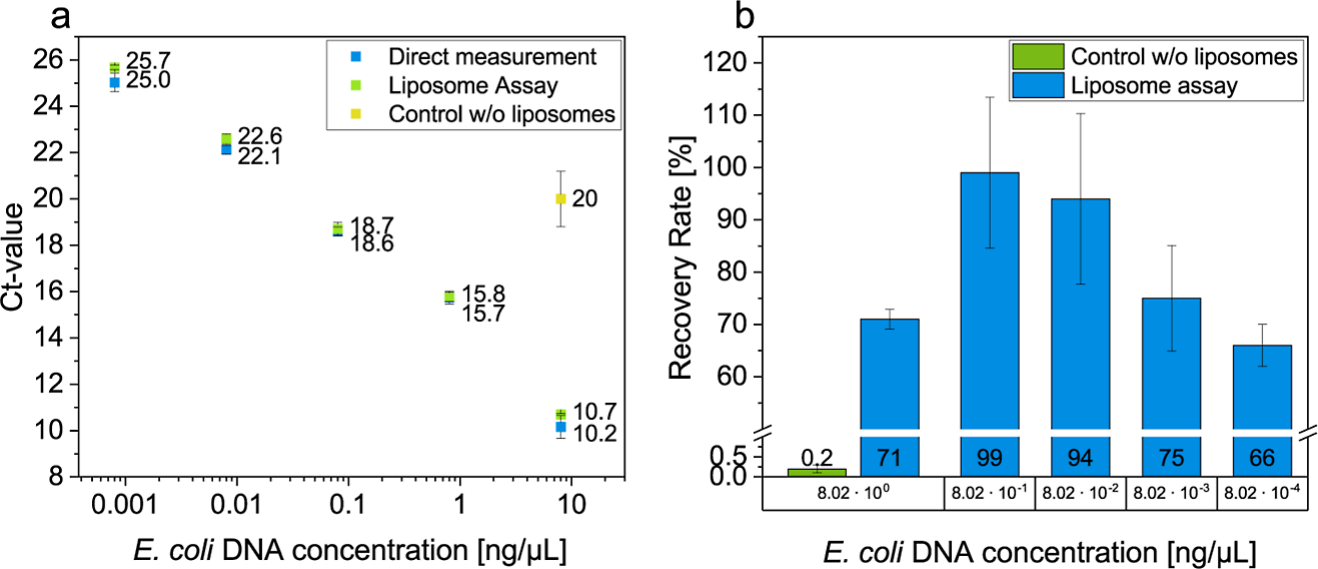
(a) Comparison of the Ct-values of the direct measurement and the liposome assay of different *E. coli* DNA concentrations. Blue: direct measurement of the *E. coli* DNA amount by adding the DNA directly into the PCR mix (n = 3). Green: liposome assay in TE buffer using cationic liposomes (n = 3). Yellow: control sample, which was processed in the same manner as the liposome samples, but did not contain any liposomes. (b) Calculated recovery rate using eqs 1 and 2 in relation to the added *E. coli* DNA concentration. Green: recovery rate of the control sample, where the protocol was carried out without liposomes (n = 3). Blue: recovery rates of the liposome assay in TE buffer (*n* = 3).

A general liposome-based protocol for DNA extraction and preconcentration was developed in which a DNA-containing sample was simply mixed with liposomes. After a short incubation, the formed lipoplex was separated by centrifugation and resolubilized in a detergent containing PCR-mix (Scheme 1). The liposome assay protocol was optimized regarding liposome concentration, incubation temperature and time, centrifugation speed and time, resuspension method, and liposome lysis (Figures S1–S5). Subsequently, the recovery rate was determined under optimal conditions by comparing the DNA content after the concentration by cationic liposomes with a qPCR measurement of the same initial DNA concentrations that was directly added to the PCR mixture (Figure 1). A control was employed in which the liposome protocol was carried out without liposomes.

High recovery rates at all DNA concentrations were obtained, with a minimum of 66% and a maximum of 99% at *E. coli* DNA concentration of 8.02×10^–4^ and 8.02×10^–1^ ng/μL, respectively. No clear dependence of the recovery rate and DNA concentration was evident. However, it could be postulated that the low recovery rate at the highest DNA concentration indicates an overload of the system, and more liposomes would have had to be added. In contrast, at very low DNA concentrations, a lower liposome concentration could have been beneficial to ensure better lipoplex formation, which leads to efficient precipitation under centrifugal forces. Too many liposomes may result in too small of lipoplexes. The comparison with the control without liposomes (0.2% recovery rate) proves the concept of extraction and preconcentration by the liposomes. Following, the liposome assay is applied to actual bacteria and viruses and is compared with commercially available kits based on SPE.

### Comparison of the Liposome Assay and Solid-Phase Extraction Regarding Extraction Efficiency for *E. coli*, *S. aureus,* and Adenovirus

**Figure 2:**
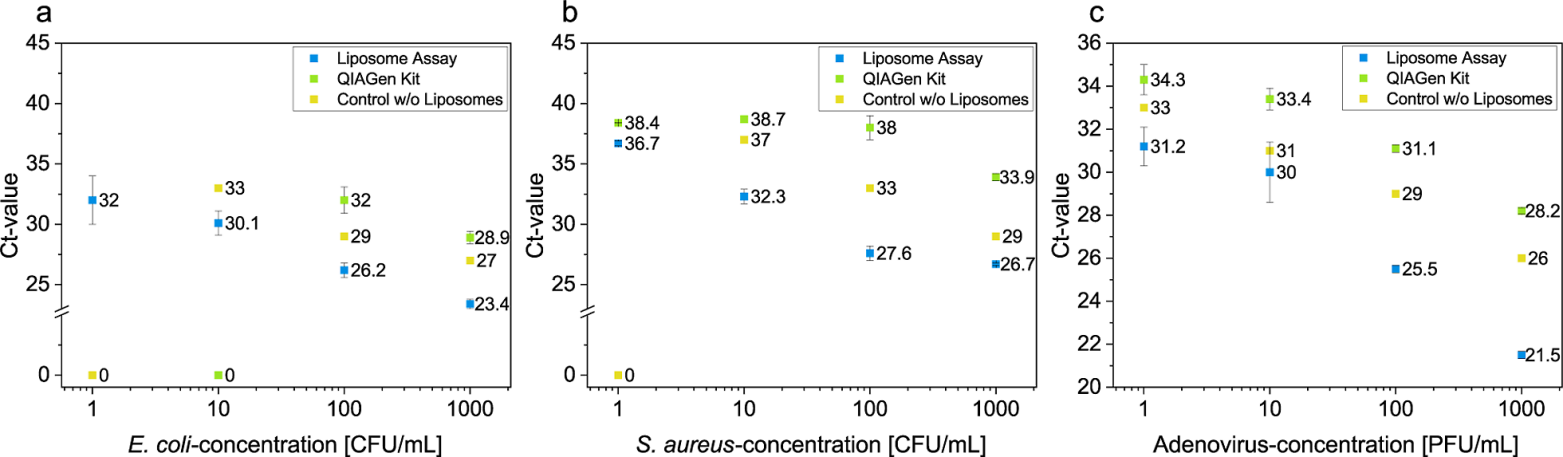
Comparison of the liposome assay and the QIAGen kit with respect to the efficiency of DNA isolation from low bacterial and virus concentrations, respectively. (a) Dose–response measurement of the liposome assay and the QIAamp DNA Mini and blood for different *E. coli* concentrations (1000, 100, 10, and 1 CFU/mL) in TE buffer. (b) Dose–response measurement of the liposome assay and the QIAamp DNA Mini and blood for different *S. aureus* concentrations (1000, 100, 10, and 1 CFU/mL) in TE buffer. (c) Dose–response measurement of the liposome assay and the QIAamp MinElute Virus Spin for different adenovirus concentrations (1000, 100, 10, and 1 PFU/mL) in TE buffer. Blue: liposome assay (n = 3), green: DNA extraction with the QIAGen kit (n = 3), and yellow: control samples of the liposome assay; the liposomes were omitted (n = 1).

The optimized liposome protocol was applied for *E. coli*, *S. aureus*, and adenovirus, as typical examples of gram-negative, gram-positive bacteria, and DNA viruses, respectively. The general liposome protocol was applied, including microorganism lysis and simple lysate mixture with liposomes. *E. coli* was lysed via sonication, and *S. aureus* and adenovirus were lysed using bead beating. As a control, the liposome protocol was carried out without liposomes. For comparison, QIAGen kits (QIAamp DNA Mini and Blood or QIAamp MinElute Virus Spin) were selected as a known example of SPE kits based on the reversible binding of DNA to silica membranes. The kits were run with the same pathogen concentrations as the liposome assay, and the eluted DNA was added to a PCR mix of the same composition and measured by qPCR (Figure 2). The Ct-values of the two methods at decreasing pathogen concentrations were compared to determine the detection limit.

A higher extraction efficiency was achieved with the liposome assay compared to the QIAGen kits for all concentrations and all microorganisms.

For the liposome assay and its control samples, a correlation between concentration and Ct-value can be observed in all cases, enabling a semiquantitative analysis. Using the liposome assay, pathogen concentrations down to 1 colony forming unit (CFU)/mL or plaque forming unit (PFU)/mL were detectable, respectively. The QIAGen kit was only able to detect a concentration of 100 CFU/mL for *E. coli*. In the case of *S. aureus* and adenoviruses, the kit also detected 1 CFU/mL and 1 PFU/mL, respectively, but with higher Ct-values than those of the liposome assay. This suggests that the SPE is indeed less efficient at low DNA concentrations and the associated dilution of the DNA leads to increased Ct-values. It further proves the hypothesis that the direct addition of DNA to the PCR-mix using liposomes and the resulting preconcentration leads to lower detection limits.

### Application of the Liposome Assay for the Detection of Bacteria in Water and Food Samples

**Figure 3:**
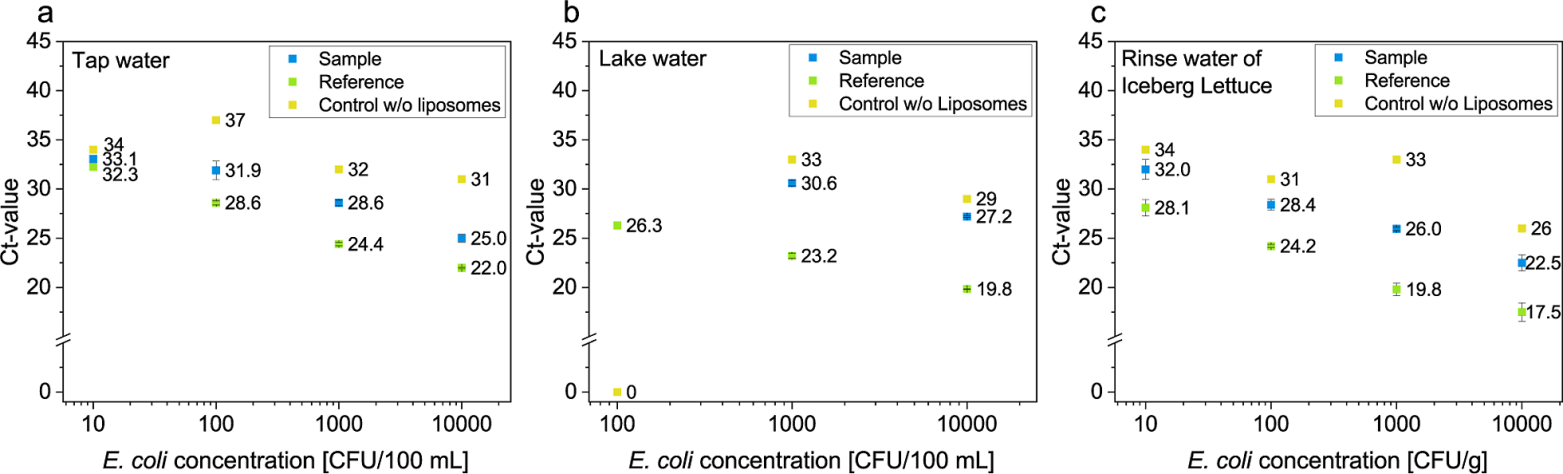
Dose–response measurement of *E. coli* bacteria concentrations in water and food samples. Particles were removed using filtration through a syringe filter (glass wool, pore size = 2 μm). Bacteria were preconcentrated by filtration through a membrane (nitrocellulose, pore size = 0.45 μm). DNA extraction was performed using cationic liposomes. (a) Tap water (100 mL) spiked with *E. coli* (10,000, 1000, 100, and 10 CFU), (b) lake water (100 mL) spiked with *E. coli* (10,000, 1000, and 100 CFU), and (c) rinse water (20 mL) of iceberg lettuce (10 g) spiked with *E. coli* (10,000, 1000, 100, and 10 CFU/g). Blue: liposome assay was performed with liposomes (n = 3). Green: reference, the same amount of bacteria was measured in TE buffer (n = 2). Yellow: control samples, the assay was performed without the addition of liposomes (n = 1).

The liposome assay was applied to tap water, lake water, and rinse water from iceberg lettuce as exemplary high-volume real samples. The samples were spiked with different concentrations of *E. coli*. In addition, a negative control without bacteria was measured for each real sample, ensuring that any initial sample contamination with *E. coli* was effectively excluded. To eliminate larger particles, filtration through a syringe filter (glass wool, pore size = 2 μm) was carried out. Furthermore, the bacteria were preconcentrated out of the large volumes by filtration through nitrocellulose (pore size = 0.45 μm). The bacteria were subsequently redissolved in a smaller volume of TE buffer by tapping and a certain amount of the buffer was used for the liposome assay (Figure 3).

In order to serve as a control, the same protocol was carried out in the absence of liposomes. As a reference, the bacteria were equally diluted in TE buffer without filtration, thus ensuring that the highest possible concentration of bacteria without inhibitors can be found in the reference.

The detection of *E. coli* bacteria was successful in all real samples. The comparison between the samples with liposomes and the controls devoid of liposomes also indicated that the preconcentration of DNA by liposomes was successful and effective. Moreover, an increase in the Ct-values of the real samples compared to the reference in TE buffer is evident, which is presumably attributable to bacterial loss during filtration. The nature of the sample is a significant factor, noticeable when comparing the results of the reference and real samples of tap water and lake water, where the Ct-value difference increases from an average of 3.5 to 7.35 units. This increase is likely due to the presence of interfering substances that continue to be part of the sample. Nontarget DNA can be excluded as an interfering substance, as evidenced by the results of the interference study involving low concentrations of *E. coli* and high amounts of *S. aureus* bacteria (Figure S9). The detection limits were determined as the concentrations at which no false-negative results were obtained. For tap water, concentrations of 10 CFU in 100 mL of water were also detectable, but one sample of the reference and two samples of the real samples are false-negative. This can be explained by the loss of bacteria during filtration or the dilution of the sample. Overall, the obtained detection limits of *E. coli* in the real samples are 100 CFU/100 mL tap water, 1000 CFU/100 mL lake water, and 100 CFU/10 g iceberg lettuce. The development of extended washing procedures may further reduce these limits of detection in the future.

### MagBead Assay Recovery Rate Determination

**Scheme 2:**
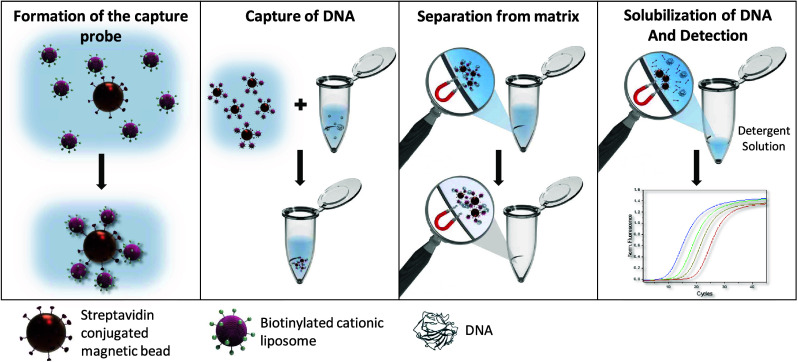
Schematic of the Working Principle of the magBead Assay

For the development of a nucleic acid isolation technology based on cationic liposomes but bypassing centrifugation, magnetic beads were used as a simple separation method for the lipoplex. The interaction between the magBeads and the liposomes is based on biotin–streptavidin binding (Scheme 2).

The purchased magBeads were conjugated with streptavidin on the surface. Biotinylated cationic liposomes were synthesized according to the previously used protocol.^28^ In this case, 18 Mol% EDPPC and 2 Mol% DPPE-biotin were used to easily modify the liposomes for their intended application. The liposomes were fully characterized in terms of tL amount, size, and ζ-potential (Table S2) to allow for reliable comparison between synthesis lots.

A magBead protocol was developed in which liposomes and the magBeads were first preincubated to form a capture probe for DNA, based on the electrostatic interaction of liposomes and DNA.

**Figure 4:**
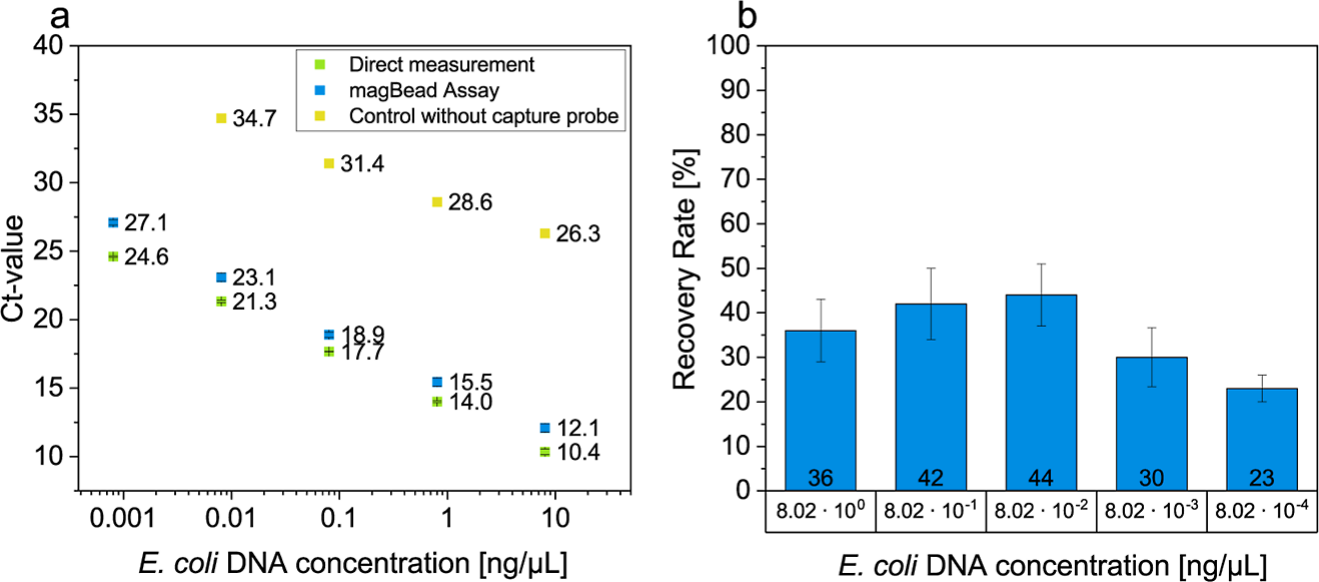
(a) Comparison of the Ct-values of different *E. coli* DNA concentrations using the direct measurement and the preconcentration assay with streptavidinylated magBeads and biotinylated cationic liposomes. Green: direct measurement of the *E. coli* DNA amount by adding the DNA directly into the PCR mix (n = 2). Blue: MagBead assay in TE buffer using cationic liposomes and streptavidinylated magBeads (n = 3). Yellow: control samples of the magnetic bead assay by omitting the magBead-liposome complex (n = 1). (b) Calculated recovery rate using eqs 1 and 2 depending on the added *E. coli* DNA concentration (n = 3).

This magBead-liposome capture probe was mixed with a DNA-containing sample. After incubation, the capture probe was separated from the rest of the sample via a magnet. As before, the DNA was released from the capture probe by specifically lysing the liposomes through heat and detergent. The detergent solution containing the dissolved DNA and lysed liposomes can again be added completely to the PCR mix without measurement interference. This magBead assay protocol was optimized regarding capture probe and sample volume, incubation time, and liposome lysis (Figures S6–S8). Subsequently, the recovery rate was determined under optimal conditions by comparing it to a qPCR measurement of the same DNA concentrations that had not undergone the DNA extraction protocol (Figure 4). In order to serve as a control, the same protocol was carried out in the absence of a capture probe.

Moderate recovery rates at all DNA concentrations were obtained, with a minimum of 23% and a maximum of 44% at *E. coli* DNA concentration of 8.02×10^–4^ and 8.02×10^–2^ ng/μL, respectively. This is significantly lower compared to yields up to 99% obtained with the centrifugal assay protocol. This may be due to significantly reduced diffusion rates of the large magBeads. Surprisingly, no clear dependence of the recovery rate and DNA concentration was evident. At high concentrations, overloading of the system can be ruled out, as even higher capture sample quantities did not lead to improved recovery (Figure S7a). Yet, similarly to the centrifugation approach, at low DNA concentrations, it can be assumed that the probability of the capture probe and DNA interaction was minimized and therefore led to a worse recovery rate. The comparison with the controls without the capture probe (0% recovery rate) proves the concept of extraction and preconcentration by the magBead-liposome complex.

### Comparison of the magBead Assay and the Liposome Assay Regarding Their DNA Extraction Efficiency from *E. coli*

**Figure 5:**
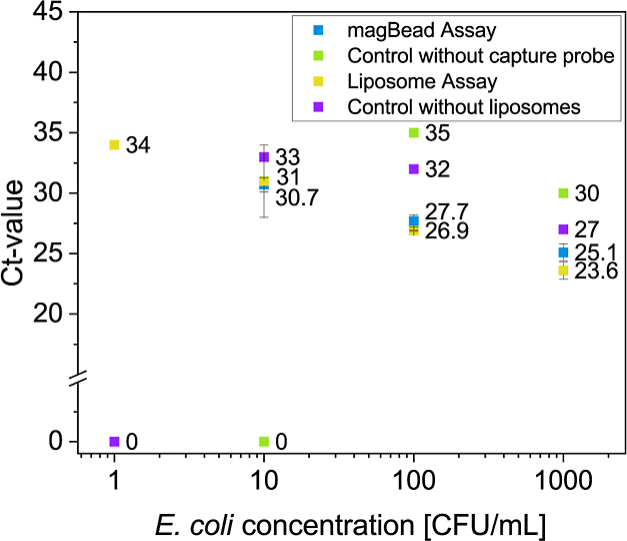
Comparison of the liposome assays and the magBead assay regarding their extraction efficiency of DNA from gram-negative bacteria. Dose–response measurements of the DNA extraction assays for different *E. coli* concentrations (1000, 100, 10, and 1 CFU/mL) in TE buffer. Blue: MagBead assay using the liposome-magnetic bead capture probe as the separation method (n = 3). Green: control of the magBead assay lacking the capture probe (n = 1). Yellow: liposome assay using centrifugation as separation method for the lipoplex (n = 2). Purple: control of the liposome assay by lacking the liposomes (n = 1).

The optimized magBead protocol was applied for different concentrations of *E. coli* bacteria, where the bacteria were lysed and the lysate mixed with the magBead-liposome capture probe. For comparison, the same amount of bacteria was treated with the liposome assay using centrifugation as the separation method. As controls, the protocols were carried out without the capture probe or the liposomes, respectively. The Ct-values of the two methods at increasing *E. coli* concentrations were compared to determine the extraction efficiency and the detection limit (Figure 5).

The magBead assay demonstrated the capacity to detect 10 CFU/mL, a notable improvement over the 100 CFU/mL detection limit achieved without the capture probe. As expected, using centrifugation, lower Ct-values for almost all bacteria concentrations and a lower detection limit of 1 CFU/mL was achieved than with the magBead approach. This result is consistent with the determination of the recovery rate, which was on average 48% higher with the liposome assay, including centrifugation. However, it is also noticeable that the controls of the liposome assay consistently show 3 units lower Ct-values compared to the magBead assays. This indicates that centrifugation either increases the nonspecific binding of DNA to the reaction tubes or that the fraction of unlysed bacteria is centrifuged off and enters the PCR-mix. It is therefore possible that both methods have approximately the same efficiency but differ in their background signal, which results in the liposome assay having a lower detection limit. In conclusion, both methods can be used effectively to improve the detection of pathogen samples exhibiting low concentrations.

## Conclusions

We studied a whole new concept of vanishing solid surface DNA extraction and demonstrated it successfully with a proof of principle. The principal advantage of liposomes is the concentration of DNA in a sample. In just a few steps, the complete DNA of a sample can be added to an amplification solution, thereby greatly reducing the detection limits. Unlike solid surfaces such as silica or chitosan and other polymers, there are limited washing strategies available due to the need to avoid lysis of liposomes. This restricts the use of organic solvents, chaotropic salts, and detergents in the lysing and extraction protocol. While this method may therefore have its limitations with highly contaminated samples requiring such stringent and harsh conditions, it is important to realize that these conditions can potentially damage the nucleic acids and be hazardous to human health.^29,30^ The extraction with cationic liposomes is not suggested to be applied as the ultimate method for all applications but rather for use in trace analysis to take advantage of the vanishing surface effect. Surprisingly, liposomes remain stable even with low detergent and solvent concentrations as long as the osmolality is maintained.

In the future, strategies should be investigated that avoid the centrifugal step in order to simplify the extraction process. One promising approach is the use of magnetic beads, as investigated in this study. However, it was found that this method did not match the extraction efficiency of the centrifugation assay, likely due to the slower diffusion rate of the capture probe. Further research in this field is therefore necessary, for example, by combining the two components directly into magnetic liposomes,^31^ using filtration techniques,^32^ or a heterogeneous assay with immobilized streptavidin to capture biotinylated lipoplexes. These strategies would also have potential for application in microfluidic systems, which are known for their suitability for point-of-need applications.^33^ In addition, this method could also be applied to biological fluids, such as mouth swabs or earwax, with the advantage that only small sample volumes are required, making it ideal for infant screening purposes. Nevertheless, the considerable presence of charged molecules in quantities exceeding those of DNA necessitates the implementation of a sample preparation procedure, such as protein aggregation, to address this issue.

## Supporting Information Available

Buffer composition, liposome synthesis and characterization, qPCR primers and measurement setup, parameter optimization of the liposome assay (e.g., lysing method, incubation temperature, liposome concentration, incubation time, resuspension method, centrifugation time, and force), parameter optimization of the magBead assay (e.g., incubation time, capture probe volume, sample volume, and liposome lysis), and an interference study about the impact of a high amount of nontarget bacteria on the extraction efficiency (PDF)
