# Supplementary material for: Studying Cationic Liposomes for Quick, Simple, and Effective Nucleic Acid Preconcentration and Isolation
Source: Anal Chem. 2025 Jan 29;97(11):6018–26. doi: 10.1021/acs.analchem.4c05936 (PMC11948181; doi:10.1021/acs.analchem.4c05936)
Supplement: Supplementary file 1 — ac4c05936_si_001.pdf [file ac4c05936_si_001.pdf]

Supporting information

Studying cationic liposomes for quick, simple, and effective nucleic acid pre-concentration and isolation.

Rahel Gruenberger[a], Changyoon Baek[b], Clemens Spitzenberg[a], Junhong Min[b*], Antje J. Baeumner[a*]

[a] Institute of Analytical Chemistry, Chemo- and Biosensors, University of Regensburg, Universitaetsstr. 31, 93053 Regensburg, Germany

[b] School of Integrative Engineering, Chung-Ang University, Heukseok-Dong, Dongjak-Gu, Seoul 06974, Republic of Korea

[a*] antje.baeumner@ur.de, Phone: +49 941 943 4065, Postal address: Universität Regensburg, 93040 Regensburg, Germany, Orcid-ID: 0000-0001-7148-3423

[b*] junmin@cau.ac.kr, Phone: +82 10 8998 0885, Postal address: Chung-Ang University, 06974, Seoul Republic of Korea, Orcid-ID: 0000-0001-9410-8204

Table of Content

Supporting information 1

Studying cationic liposomes for quick, simple, and effective nucleic acid pre-concentration and isolation. 1

Experimental section 3

Buffer composition 3

Medium composition 3

Liposome Characterization 3

PCR-measurement 3

Parameter optimization of the liposome assay 4

Lysing method 4

Incubation temperature and liposome concentration 5

Incubation Time 6

Resuspension method 7

Centrifugation time and force 8

Parameter optimization of the magBead assay 9

Incubation time 9

Capture probe 10

Liposome lysis 11

Interference study of *E. coli* with *S. aureus* 13

Experimental section

Buffer composition

TE buffer (pH 8) contained 10 mM Tris-(hydroxymethyl)-aminomethan and 1 mM Ethylenediaminetetraacetic acid disodium salt dihydrate (EDTA). The buffer was filtered with a with a Nylone Membrane (0.20 µm, hydrophilic).

HSS buffer (pH 7.5) contained 10 mM HEPES, 200 mM NaCl, 200 mM sucrose and 0.01 % $NaN_{3.}$

Binding and Washing buffer (pH 7.5) contained 0.5 mM EDTA,1 M NaCl in 5 mM Tris-HCl.

Medium composition

10 mg of lysogen broth (LB) was solved in 500 mL bidest. water. Before usage, the medium was autoclaved for 20 min. The medium was stored in the refrigerator.

Liposome Characterization

Table S1: Parameters (Lipid composition, Encapsulant, ζ-Potential, Z-average, Polydispersity Index (PdI) and total lipid) of the used liposomes

	**Lipid composition**	**Encapsulant**	**ζ-potential [mV]**	**Z-average [µm]**	**PdI**	**Total lipid [mM]**
**Cationic liposomes**	76.7 Mol% DPPC<br>18.1 Mol% EDPPC<br>5.2 Mol% Cholesterol	300 mM NaCl	14 ± 2	220 ± 5	0.235 ± 0.0006	12.03 ± 0.04
**Biotinylated cationic liposomes**	75 Mol% DPPC<br>18 Mol% EDPPC<br>5 Mol% Cholesterol<br>2 Mol% DPPE-Biotin	300 mM NaCl	13.5 ±0.3	199 ± 7	0.246 ± 0.013	10.81 ± 0.04

PCR-measurement

Table S2: Reverse and forward primer sequences for E. coli bacteria, S. aureus bacteria and Adenovirus

E. coli	**Forward**	5'-ACT TCG ACA AAT ATG CTG GC-3'
	**Reverse**	5'-CGG GAT GAT GTT CTG GGA A-3'
S. aureus	**Forward**	5'-GTT GCA TCG GAA ACA TTG TGT-3'
	**Reverse**	5'-ATG ACC AGC TTC GGT ACT ACT AAA GAT-3'
**Adenovirus**	**Forward**	5'-GGT GTC GCG CTT GCC TAC TA-3'
	**Reverse**	5'-CGA TCG CGT TGT TCA TAA G-3'

Table S3: Measurement protocol for the real-time PCR in the Qiagen Rotor Gene Q

	**Cycles**	**Temperature [°C]**	**Time**
**Pre-Incubation**	1	95	5 min
**Amplification**	45	95	10 s
		60	10 s
		72	10 s
**Melting Curve**	1	65 to 95	1 min/°C

Parameter optimization of the liposome assay

The liposome assay was optimized regarding lysis, incubation parameters, resuspension, and centrifugation parameters by varying the parameters and comparing the Ct-values of the real-time PCR.

Lysing method

The liposomes were lysed using various methods to determine the influence of intact liposomes on qPCR. For this purpose, a liposome dilution was prepared in TE buffer (0.9 mM, 100 µL) and lysed. *E. coli* DNA was added, and 2 µL of the resulting solution was combined with an '18 µL'-PCR mix for measurement (Figure S1). A positive control containing DNA without liposomes was measured. As a control whether intact liposomes have an influence on the PCR, one sample was not lysed. Three lysis methods were investigated: heating at 90°C for 5 min, addition of Tween80 (10 vol%) for 5 min at room temperature and a combination of both methods involving Tween80 (10 vol%) for 5 min at room temperature and heating at 85°C for 5 min.

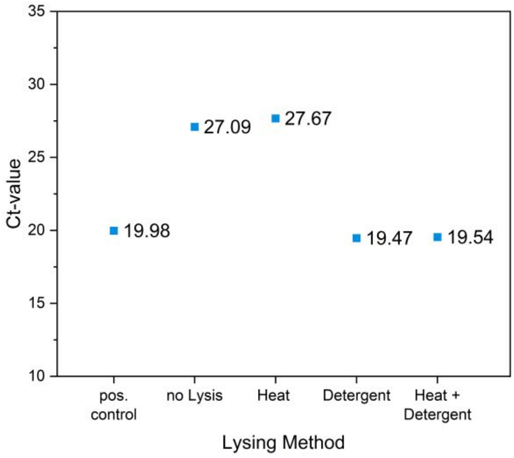

Figure S1: Comparison of different lysing methods of the liposomes (0.9 mM total lipid content) prior to DNA addition (0.062 ng/µL) and qPCR measurement (n = 1). 2 µL of the resulting solution were added to the PCR mix.

- ***Positive control:** DNA dilution in TE-buffer without liposomes.*
- ***no Lysis: L**iposomes were not treated with a lysing method.*
- ***Heat:** Liposomes were lysed by heating up to 90°C for 5 min.*
- ***Detergent:** Liposomes were lysed by adding 10 vol%. Tween80 for 5 min at room temperature.*
- ***Heat + Detergent:** Liposomes were lysed by adding 10 vol%. Tween80 for 5 min at room temperature and an additional heating step for 5 min at 85 °C.*

Comparison of the two controls, revealed that intact liposomes interfere with qPCR. The sample without liposomes has a significantly lower Ct-value than the sample with intact liposomes (7 units). This is probably caused by the DNA being tightly bound to the liposomes or embedded between the lipid bilayers and preventing access for the DNA polymerase for amplification. Heating to 90°C does not appear to provide efficient lysis, as a similar value to the control with intact liposomes is achieved. The most efficient lysis methods appear to be the addition of a detergent and the combination of both methods, as these achieve similar values to the positive control. The lysis efficiency of the combination method is probably due to the detergent, as this method alone works equally well. For this reason, only lysis with Tween80 is used in the following, as an additional heating and sample preparation step is not necessary.

Incubation temperature and liposome concentration

The liposome assay was conducted for *E. coli* DNA (49.44 ng/mL) using different liposome concentrations (90 µM and 9 µM) at various incubation temperatures (room temperature, 30°C and 40°C) for 15 min. The lipoplex was centrifuged at 15000 g for 15 min and resolubilized in TE buffer (100 µL). 2 µL of the resulting solution was combined with an '18 µL'-PCR mix for measurement (Figure S2Figure S1). A positive control was prepared by diluting the same amount of DNA in TE buffer (100 µL) and adding 2 µL to an '18 µL'-PCR mix.

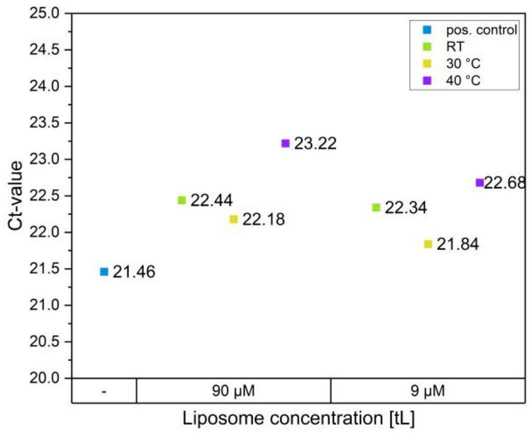

Figure S2: Comparison of different liposome assay variations in incubation temperature and total lipid content. Temperature for incubation was varied from room temperature to 40 °C. Total lipid content was varied between 90 µM and 9 µM total lipids content prior to centrifugation. An incubation time of 15 min was used and centrifugation for 15 min at 15000 g, the lipoplex was resolubilized in TE-buffer (100 µL) and added afterwards into a PCR-mix containing Tween80 (4 vol%). (n = 1)

For both liposome concentrations, the same trend can be observed in the efficiency of the assay in relation to the incubation temperature. Incubation at 30°C shows the lowest Ct-value, at room temperature a slightly higher Ct-value is achieved and at 40°C the worst efficiency is obtained. Comparing the Ct-values of the two liposome concentrations, there is also a clear trend showing that 9 µM achieves the better efficiency.

Therefore, the liposome concentration of 9 µM and the incubation temperature of 30°C are used as optimal conditions, as this combination showed the Ct-value with the smallest difference to the positive control (0.38 units).

Incubation Time

The liposome assay using cationic liposomes (9 µM) to capture *E. coli* DNA (24.72 ng/mL) was conducted using various incubation times (5 min, 15 min, 30 min and 60 min) at 30°C. The formed lipoplex was centrifuged at 15000 g for 15 min and resolubilized in TE buffer (100 µL). 2 µL of the resulting solution was combined with an '18 µL'-PCR mix for measurement (Figure S3Figure S2Figure S1). A positive control was prepared by diluting the same amount of DNA in TE buffer (100 µL) and adding 2 µL to an '18 µL'-PCR mix.

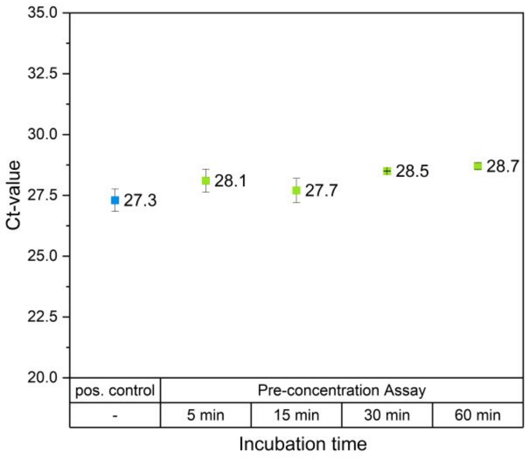

Figure S3: Comparison of varying incubation times (5 min, 15 min, 30 min and 60 min) at a set DNA concentration (24.72 ng/mL). The total lipid concentration in the sample was set to 9 µM an incubation temperature of 30 °C was used and centrifugation for 15 min at 15000 g, the lipoplex was resolubilized in TE-buffer (100 µL) and added afterwards into a PCR-mix containing Tween80 (4 vol%). (n = 2)

Comparing the Ct-values a similar extraction efficiency is evident for most incubation times. The sample with an incubation time of 15 min shows the minimum Ct-value with the lowest difference to the positive control (0.4 units).

Resuspension method

The liposome assay using cationic liposomes to capture *E. coli* DNA was conducted. The total lipid concentration in the sample was set to 9 µM an incubation temperature of 30 °C was used for 15 min. The formed lipoplex was centrifuged of for 15 min at 15000 g and resolubilized using two different methods. The lipoplex was either resuspended in TE buffer (100 µL) and afterwards added (2 µL) to an '18 µL'-PCR-mix or directly resuspended in a '20 µL'-PCR mix. Both methods were then compared to the corresponding positive control, where the DNA dilution (2 µL) was directly added to the PCR-mix and the recovery rate was determined (Figure S4). A negative control was measured where the liposomes were omitted, and the DNA was resuspended in TE buffer (100 µL).

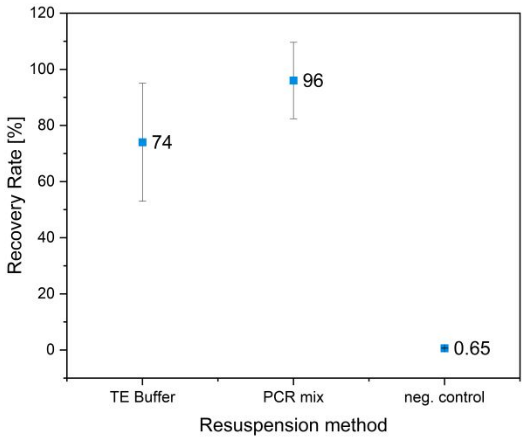

Figure S4: Comparison of the recovery rate of two solubilization methods of the lipoplex at different DNA concentrations. The lipoplex was either resuspended in TE buffer (100 µL) and afterwards added to the PCR-mix (DNA concentration: 24.72 ng/mL) or directly resuspended in the PCR-mix (DNA concentration: 123.6 ng/mL). Both methods were then compared to the corresponding positive control, where the DNA dilution was directly added to the PCR-mix. A negative control was measured where the liposomes were omitted, and the DNA was resuspended in TE buffer. The total lipid concentration in the sample was set to 9 µM an incubation temperature of 30 °C was used for 15 min and it was centrifuged for 15 min at 15000 g. (n = 2)

The recovery rates of the two resuspension methods show high efficiency in both cases. However, direct dissolution in the PCR mix is very effective with a recovery rate of 96 % and additionally saves a pipetting and dilution step, which subsequently leads to easier handling and lower detection limits.

Centrifugation time and force

The liposome assay using cationic liposomes (9 µM) to capture *E. coli* DNA (0.124 ng/mL) was conducted using an incubation temperature of 30 °C for 15 min. The centrifugation parameters were optimized by varying centrifugation time (10 min, 20 min and 30 min) and centrifugation force (7,500 g, 15,000 g and 20,000 g). The formed lipoplex was resolubilized directly in a '20 µL'-PCR mix for measurement (Figure S5Figure S2Figure S1).

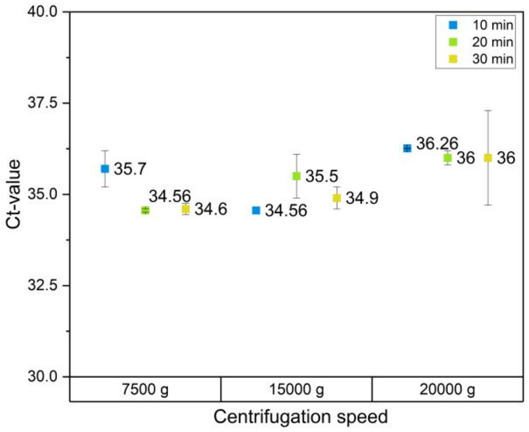

Figure S5: Comparison of the liposome assay variation in centrifugation time (10 min, 20 min and 30 min) and speed (7,500 g, 15,000 g, and 20,000 g). The assay was conducted using a DNA concentration of 0.124 ng/mL. The total lipid concentration in the sample was set to 9 µM an incubation temperature of 30 °C was used for 15 min, the lipoplex was resolubilized directly in the PCR-mix (20 µL) containing Tween80 (4 vol%). (n = 2)

A comparison of the Ct values of the different parameter variants shows that a centrifugation speed of 20,000 g has the worst efficiency. The high centrifugal force probably causes the liposomes to disrupt, leading to premature DNA release. At the parameter combinations of 7,500 g and 20 min or 30 min centrifugation time and at 15,000 g and 10 min, the Ct values are similar. However, with the higher centrifugation force of 15,000 g, the time can be reduced to 10 min, which leads to a shortening of the assay time. This combination is therefore considered optimal.

Parameter optimization of the magBead assay

The magBead assay was optimized regarding incubation time, capture probe volume, sample volume and lysing conditions by varying the parameters and comparing the Ct-values of the real-time PCR.

Incubation time

The magBead assay using the cationic liposomes-magBead capture probe (5 µL) to capture *E. coli* DNA (0.802 ng/µL) in a TE buffer solution (500 µL) was conducted using various incubation times (30 min, 45 min and 60 min) at 30°C using a thermos shaker (300 rpm). The formed DNA-liposome-magBead complex was captured using a magnet and washed with TE buffer (100 µL). The capture probe was resolubilized in a detergent solution Tween80 (4 wt%, 5 µL) for 10 min at room temperature to lyse the liposomes and release the DNA. The entire detergent solution was then added into a '15 µL'-PCR-mix (n = 3). As reference the same DNA amount (2 µL) was directly spiked into a '18 µL'-PCR-mix (n = 2). The Ct-values of the assays with different incubation parameters are compared to evaluate the optimal incubation time for DNA extraction (Figure S6).

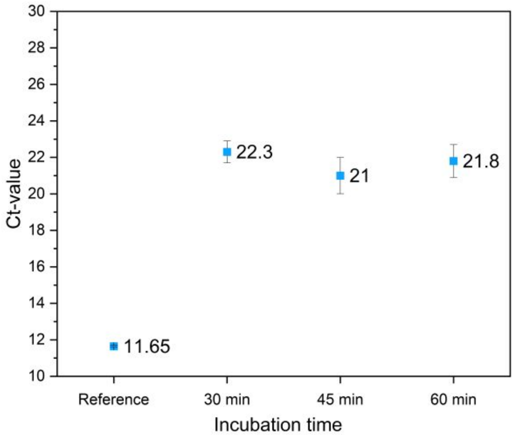

Figure S6: Comparison of varying incubation times (30 min, 45 min and 60 min) at a set E. coli *DNA concentration (0.802 ng/µL). The total capture probe volume was set to 5 µL in a sample volume of 500 µL. An incubation temperature of 30 °C was used. The formed DNA-liposome-magBead complex was captured using a magnet, the supernatant was removed and the capture probe was washed with TE buffer (100 µL). The capture probe was resolubilized in Tween80 (4 wt%, 5 µL) for 10 min at room temperature and the detergent solution with the solubilized DNA was added afterwards into a '15 µL'-PCR-mix (n = 3). As reference the same DNA amount (2 µL) was directly spiked into a '18 µL'-PCR-mix (n = 2).*

A comparison of the Ct values shows a similar extraction efficiency for most incubation times. The sample with an incubation time of 45 minutes shows the lowest Ct value with the smallest difference to the positive control (9.35 units). However, it can be seen that further optimization is necessary to achieve a better extraction efficiency.

Capture probe

The magBead assay using the cationic liposomes-magBead capture probe to capture *E. coli* DNA in a TE buffer solution was conducted using various capture probe volumes and sample volumes. The samples were incubated for 45 min at 30°C using a thermos shaker (300 rpm). The formed DNA-liposome-magBead complex was captured using a magnet. The capture probe was resolubilized in a detergent solution. For the capture probe optimization Tween80 (4 wt%, 5 µL) was used for 10 min to lyse the liposomes and release the DNA. For the sample volume optimization OG (10 mM, 5 µL) for 10 min at 90°C was used. The entire detergent solution was then added into a ’15 µL’-PCR-mix (n = 3). As reference the same DNA amount (2 µL, 0.802 ng/µL) was directly spiked into a ’18 µL’-PCR-mix (n = 2). The Ct-values of the assays with different capture probe and sample volumes are compared to evaluate the optimal parameters for DNA extraction (Figure S7).

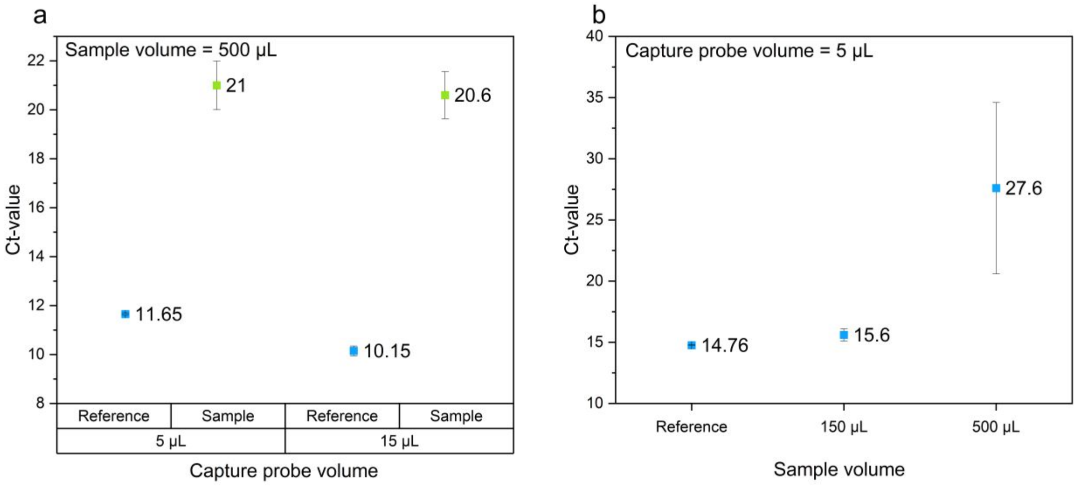

Figure S7: Comparison of the Ct-values of magBead assay using different capture probe to sample volume ratios to references with the same DNA amount (2 µL, 0.802 ng/µL) directly spiked into a ’18 µL’-PCR-mix (n = 2).

a) Variation of the capture probe volume (5 µL and 15 µL) in a DNA containing sample volume of 500 µL. The liposomes were lysed using Tween80 (4 wt%, 5 µL) for 10 min at room temperature.

b) Variation of the sample probe volume (150 µL and 500 µL) containing the same amount of DNA. The capture probe volume was set to 5 µL. The liposomes were lysed using OG (10 mM, 5 µL) for 10 min at 90°C.

The samples were incubated for 45 min at 30°C. The formed DNA-liposome-magBead complex was captured using a magnet. The capture probe was resolubilized in a detergent solution. The entire detergent solution was then added into a ’15 µL’-PCR-mix (n = 3).

A comparison of the Ct-values of the positive control to different capture probe volumes used in the magBead assay at the same sample volume of 500 µL shows no improvement in extraction efficiency by adding three times the amount of magBeads (Difference for 5 µL: 9.35 units. Difference for 15 µL: 10.45 units). However, reducing the sample volume from 500 µL to 150 µL significantly increases extraction efficiency. The magBeads have a relatively high density and tend to sink quickly, which is why they are more likely to encounter the analyte when the sample volume is reduced.

Liposome lysis

The magBead assay using the cationic liposomes-magBead capture probe (5 µL) to capture *E. coli* DNA (0.802 ng/µL) in a TE buffer solution (150 µL) was conducted using various lysing conditions. Two different detergents were evaluated for their lysing efficiency. Additionally, various lysing times (10 min and 20 min) and temperatures (room temperature, 40 °C, 90 °C) were used to efficiently solubilize the DNA. The samples were incubated with the capture probe for 45 min at 30 °C using a thermos shaker (300 rpm). The formed DNA-liposome-magBead complex was captured using a magnet. The capture probe was resolubilized in a detergent solution (5 µL) to lyse the liposomes and release the DNA. The entire detergent solution was then added into a '15 µL'-PCR-mix (n = 3). As reference the same DNA amount (2 µL) was directly spiked into a '18 µL'-PCR-mix (n = 2). Two negative controls were prepared by incubating the samples with blank magBeads without liposomes and omitting the capture probe completely (n = 2). The Ct-values of the assays with different lysing parameters are compared to evaluate the optimal elution parameters for DNA extraction (Figure S8Figure S6).

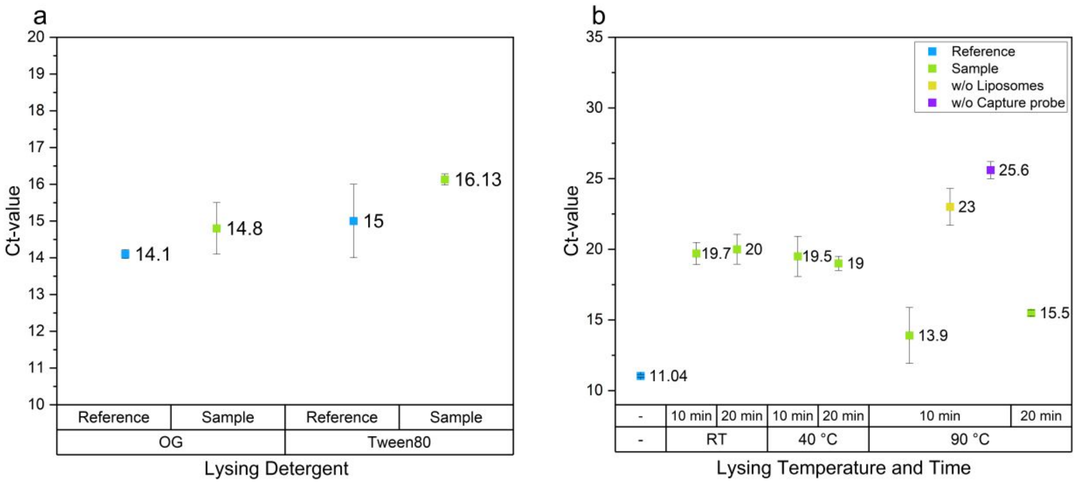

Figure S8: Comparison of the Ct-values of magBead assay using various liposome lysing conditions to references with the same DNA amount (2 µL, 0.802 ng/µL) directly spiked into a '18 µL'-PCR-mix (n = 2) to determine the most efficient parameters for DNA elution.

a) Comparison of liposome lysis using two different detergents (Tween80, 4 wt% and OG, 10 mM) at 90°C for 10 min. (n = 3)

b) Comparison of liposome lysis varying lysis time (10 min and 20 min) and temperature (room temperature, 40°C and 90°C) using OG (10 mM) (n = 3. Two negative controls were prepared by incubating the samples with blank magBeads without liposomes and omitting the capture probe completely (n = 2).

The samples (150 µL) containing E. coli *DNA (2 µL, 0.802 ng/µL) were incubated with the liposomes-magBead capture probe (5 µL) for 45 min at 30 °C using a thermos shaker (300 rpm). The formed DNA-liposome-magBead complex was captured using a magnet. The capture probe was resolubilized in a detergent solution (5 µL) to lyse the liposomes and release the DNA. The entire detergent solution was then added into a '15 µL'-PCR-mix (n = 3). As reference the same DNA amount (2 µL) was directly spiked into a '18 µL'-PCR-mix (n = 2).*

By using OG in comparison to Tween80, the respective difference to the reference could be reduced from 1.13 units to 0.7 units. The lysis of the liposomes appears to be the decisive parameter to be optimized, as this is where most of the differences can be seen when varying the temperature. An elevation of the temperature during lysis leads to a decrease in Ct-values and thus to an improved extraction efficiency, as the liposomes are increasingly lysed, and the DNA is thus resuspended. Extending the lysis time from 10 min to 20 min leads to an increase in the Ct-values at a high temperature (90°C), as the DNA is presumably denatured. The optimized parameters for DNA resuspension are therefore lysing at 90°C for 10 min.

The comparison between the capture probe samples with the negative controls with blank magBeads or without the capture probe further confirms that the DNA extraction can be attributed to the use of cationic liposomes.

Interference study of *E. coli* with *S. aureus*

The influence of a high concentration of another bacterium on the extraction efficiency of the liposome assay was investigated by preparing samples containing different concentrations of *E. coli* (1000 CFU/mL, 100 CFU/mL and 10 CFU/mL) and a constant amount of *S. aureus* (10^5 CFU/mL). As references samples containing different concentrations of *E. coli* without *S. aureus* were prepared (Figure S9). The liposome assay was conducted for all samples using the optimized parameters for *E. coli* detection.

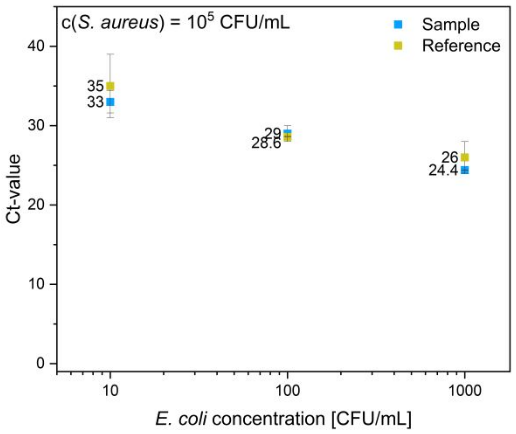

Figure S9: Comparison of the liposome assay at different concentrations of E. coli *(1000 CFU/mL, 100 CFU/mL and 10 CFU/mL) and a constant amount of S. aureus (10^5 CFU/mL) to references with only E. coli and without S. aureus, to assess the influence of the presence of a high amount of additional DNA on the extraction efficiency of the liposome assay.*

The Ct-values of the reference without additional bacteria and the samples with the interference bacterium *S. aureus* are very similar at all *E. coli* concentrations. This indicates that the extraction efficiency of the liposomes is not affected by an additional high amount of DNA. This is particularly relevant for real samples where a mixed culture of bacteria may be present in addition to the target bacterium.
